# An Efficient Hierarchical Generalized Linear Mixed Model for Mapping QTL of Ordinal Traits in Crop Cultivars

**DOI:** 10.1371/journal.pone.0059541

**Published:** 2013-04-02

**Authors:** Jian-Ying Feng, Jin Zhang, Wen-Jie Zhang, Shi-Bo Wang, Shi-Feng Han, Yuan-Ming Zhang

**Affiliations:** Section on Statistical Genomics, State Key Laboratory of Crop Genetics and Germplasm Enhancement, Department of Crop Genetics and Breeding, Nanjing Agricultural University, Nanjing, Jiangsu, China; Pennsylvania State University, United States of America

## Abstract

Many important phenotypic traits in plants are ordinal. However, relatively little is known about the methodologies for ordinal trait association studies. In this study, we proposed a hierarchical generalized linear mixed model for mapping quantitative trait locus (QTL) of ordinal traits in crop cultivars. In this model, all the main-effect QTL and QTL-by-environment interaction were treated as random, while population mean, environmental effect and population structure were fixed. In the estimation of parameters, the pseudo data normal approximation of likelihood function and empirical Bayes approach were adopted. A series of Monte Carlo simulation experiments were performed to confirm the reliability of new method. The result showed that new method works well with satisfactory statistical power and precision. The new method was also adopted to dissect the genetic basis of soybean alkaline-salt tolerance in 257 soybean cultivars obtained, by stratified random sampling, from 6 geographic ecotypes in China. As a result, 6 main-effect QTL and 3 QTL-by-environment interactions were identified.

## Introduction

Many characters of biological interest and economic importance vary in an ordinal form, i.e. disease and tolerance, but are not inherited in a simple Mendelian fashion. More importantly, they cause substantial yield loss. To decrease the loss, developing resistance cultivar is the most economic and effective way. Therefore, there is a critical need for in-depth study of methodology for mining elite alleles for ordinal traits.

During the past several decades, many attempts have been made to mine elite alleles for binary and ordinal traits. The methodologies of mapping quantitative trait loci (QTL) for discrete traits have been well established within the framework of threshold model. On the early stage, almost all the approaches are based on single QTL genetic model [1]–[7]. Later on, several methods have been proposed to simultaneously identify multiple QTL for ordinal traits [8], [9]. Recently, Bayesian methodology has been used to map multi-QTL and epistatic QTL for binary and ordinal traits [10]–[14]. However, all the above approaches are based on bi-parental segregating populations.

Many commercial inbred lines are available in crops. A large amount of elite alleles have preserved among these lines. Mining these elite alleles is the prerequisite in the integration of genetic analysis with crop breeding. Up to now, some approaches for mining elite alleles in crop cultivars have been developed [15]–[20]. All kinds of QTL can be effectively identified, elite alleles can be easily mined and novel parental combination can be effectively predicted [18]. However, these approaches in crop cultivars are for quantitative traits but not for discrete traits. As for discrete traits, too much complication comes from seemingly simple descriptions and unknown population structure meanwhile in fact the underlying biological model may be complicated. Accordingly, genetic analyses may be more challenging for discrete traits than for continuous traits. If pedigree information among these lines is known, Bayesian linkage analysis [21] and variance-components approach [22] have been presented. If the pedigree information is not known, relatively little has been known, except for Iwata et al. [23] and Hoggart et al. [24]. Although Iwata et al. [23] have developed Bayesian multilocus association analysis, the method is implemented via Markov chain Monte Carlo, and computing time becomes a major concern. Although Hoggart et al. [24] proposed simultaneous analysis of all SNPs in genome-wide association study, the method is for case-control dataset.

Multi-QTL mapping for discrete and quantitative traits is now the state-of-the-art method [18]–[20], [24], [25]. However, it is difficult to implement under the maximum-likelihood framework. At present the Bayesian method implemented via expectation-maximization algorithm [26] is specialized to handle complicated models and thus it is the ideal tool for mapping multiple QTL for ordinal trait in crop cultivars. Accordingly, in this study empirical Bayes approach of Xu [26] and the computational algorithm of Yi et al [27] were incorporated into the hierarchical generalized linear model of Yi et al [12] to map main-effect QTL (M-QTL) and QTL-by-environment (QE) interaction for ordinal traits in crop cultivars. The new method was validated by a series of Monte Carlo simulation experiments and real data analysis in soybean.

## Results

### Phenotypic variation for soybean alkaline-salt tolerance

**Figure 1:**
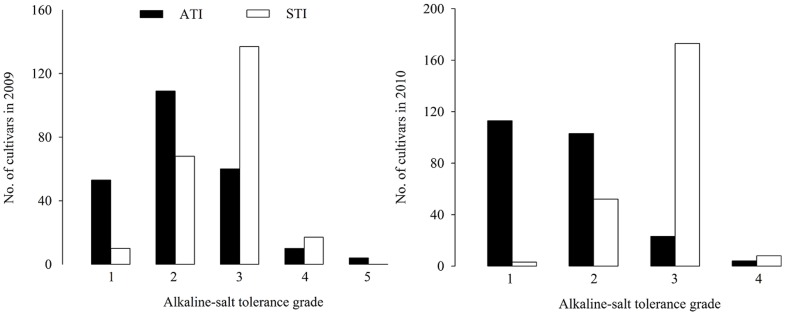
Frequency distribution for soybean alkaline-salt tolerance grade in 2009 (left) and 2010 (right).

We measured lengths of main root (LR) of 257 soybean cultivars under the cases of control (CK), 100 mM NaCl and 10 mM Na2CO3. These original trait observations might be transferred into alkaline tolerance index (ATI) and salt tolerance index (STI). To measure the degree of salt-alkaline tolerance, these indexes were partitioned into five grades: high tolerance, tolerance, middle tolerance, sensitivity, and high sensitivity. In other words, this data is ordinal. The phenotypic distributions were shown in **Fig. 1** and **Table S1**. All the two discrete indexes almost exhibited skewed distribution, indicating the existence of genetic variation. Results from 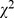 test showed that there is significant relationship between the tolerance and environment (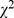 = 44.83 and *P*<1e-4 for ATI, and 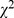 = 13.29 and *P* = 0.004 for STI), indicating the existence of environmental interaction.

### Mapping M-QTL and QE interaction for ATI and STI

A total of 6 M-QTL (3 for ATI, and 3 for STI) and 3 QE interactions (one for ATI, and 2 for STI) for soybean alkaline-salt tolerance are detected by new method, and mapped to chromosomes A1, B2, I, L, N and O. Among them, one QTL, associated with marker sat_274, is responsible simultaneously for the above two traits; seven QTL are consistent with those of continuous ATI and STI using enriched compression mixed linear model (ECMLM) [28] and epistatic association mapping (EAM) [18] methods, and the other two were also confirmed by test of independence (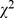 test); and one M-QTL and one QE interaction are associated simultaneously with marker satt270. A summary of all detected QTL is shown in **Table 1**.

**Table 1:** Association mapping for ordinal alkaline-salt tolerance in 257 soybean cultivars.

Trait	New method								Elite allele of detected QTL			Similar result^*^		
	QTL	Type	Chr.	Marker	Position(cM)	Variance	LOD	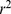 (%)	bp	Effect	Carrier	ECMLM	EAM	P(H_0_)^$^
ATI	*qati14*	MQ	14(B2)	sat_342	20.30	0.0798	6.96	5.58	260	−0.73	Zunyizongzidou	√ (MQ)		1e-4
	*qati10-1*	MQ	10(O)	satt348	15.29	0.0469	3.12	3.29	232	−0.36	Jindou 3	√ (MQ)		0.0044
	*qati10-2*	MQ	10(O)	sat_274	107.58	0.1441	3.14	10.09	412	−3.39	Ludou 1			0.0027
	*qati5e*	QE	5(A1)	sat_344	19.37	0.1578	5.07	11.04	433×2010^†^	−5.24	Baiqiu 1	√ (MQ)	√ (QE)	<1e-4
STI	*qsti20*	MQ	20(I)	satt270	50.11	0.1173	3.26	7.68	243	−1.06	Jiangechengguanbayuehuang	√ (MQ)		0.5842
	*qsti19*	MQ	19(L)	satt652	30.87	0.1401	3.23	9.17	241	−0.81	Hunanqiudou 1	√ (MQ)		0.0520
	*qsti10*	MQ	10(O)	sat_274	107.58	0.0643	4.01	4.21	394	−0.65	Baiqiu 1			0.0027
	*qsti20e*	QE	20(I)	satt270	50.11	0.1311	3.80	8.58	252×2009	−0.9771	Shuichengzongzidou		√ (QE)	0.5842
	*qsti3e*	QE	3(N)	satt022	102.05	0.0748	5.04	4.90	223×2010	−0.90	Daheiqi		√ (QE)	0.0216

MQ: main-effect QTL; QE: QTL-by-environment interaction. ^*^similar results for continuous ATI and STI were derived from **Zhang** [**33**] by enriched compression mixed linear model (ECMLM).

and epistatic association mapping (EAM) approaches. ^†^Year, i.e., 2009 and 2010. ^$^Probability of null hypothesis in the test of independence between the tolerance and marker.

4 ATI QTL, with proportion of phenotypic variance explained by single QTL (PVE) of 3.29–11.04%, are detected and mapped to chromosomes A1, B2 and O. Of these QTL, there are three M-QTL (18.96%) and one QE interaction (11.04%); and three QTL are further identified by ECMLM (or EAM) and 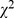 test. It should be noted that the PVE by *qAT10-2* and *qATI5e*, associated respectively with sat_274 and sat_344, are greater than 10%.

5 STI QTL, with PVE of 4.21–9.17%, are detected and mapped to chromosomes I, L, N and O. Of these QTL, there are three M-QTL (21.06%) and two QE interactions (13.48%); and all the QTL, except for *qSTI10*, are further identified by ECMLM (or EAM) and 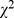 test. It should be noted that the PVE of all the QTL are less than 10%.

### Mining elite alleles

The summaries of elite allele and its representative carrier are shown in **Table 1**. As for the *qATI14* associated with Sat_342, there are 12 alleles and one unknown allele. The effects for all these alleles can be estimated by maximum likelihood method. Of these effects, the 260 bp allele has the smallest effect −0.73, being an elite allele, which can be found in soybean cultivar Zunyizongzidou. Similarly, as for the *qSTI3e* associated with satt270, the 223 bp allele shows the smallest effect in 2010, elite allele combination is the 223 bp allele×2010 with an effect of −0.90.

### Predicting novel parental combination

In a hypothetical cross of two cultivars, all the recombinant inbred lines (RILs) from the cross may be produced. In these RILs, the trait values can be predicted by the effects of all the detected loci. The best RIL with minimum value would represent the cross. Therefore, the best cross could be selected from all the crosses. It was found that any cultivar-pair does not pyramid all the elite alleles of the detected QTL. However, some four-cultivar combinations might pyramid all the elite alleles of salt-alkaline tolerances in this study, for example, the best two combinations were Zunyizongzidou × Hunanqiudou 1×Ludou 1×Qi 588-8, and Zunyizongzidou×Hunanqiudou 1×Ludou 2×Qi 588-8, which are used to simultaneously improve the two traits.

### Prediction for potential candidate genes

The summary of potential candidate genes for alkaline-salt tolerance in soybean is shown in **Table 2**. A total of 7 soybean genes homologous to *Arabidopsis* are linked to 7 markers detected in this study, with physical distances of 206.21–129132.42 kb; and one gene (Glyma03g38040) is closely linked to the associated markers (satt022) in this study, within 210 kb in physical distance.

**Table 2:** Prediction for potential candidate genes that are homologous to alkaline-salt tolerance genes in *Arabidopsis thaliana.*

Genes in Arabidopsis thaliana	Homologous genes in soybean			Associated marker in this study is around soybean homologous gene (SHG)				Marker closely linked to SHG		
	Gene	Chr.	Position (bp)	Marker 1	Position (bp)	Distance to SHG (kb)	Associated QTL	Marker 2	Distance to SHG (kb)	Distance between markers 1 & 2 (cM)
AT2G47190 (MYB2)	Glyma03g38040	3	44477360–44476292	satt022	44682505–44682712	206.21	qSTI3e	satt022	206.21	0.0
AT5G63310 (NDPK2)	Glyma05g03010	5	2300110–2296337	sat_344	3691696–3691992	1,395.36	qATI5e	Sat_368	587.14	5.01
AT5G27150 (NHX1)	Glyma10g30020	10	38712235–38706503	sat_274	43209577–43209848	4,503.07	qATI10-2, qSTI10	Sat_242	132.468	33.53
AT1G69270 (RPK1)	Glyma13g06210	10	6487011–6490433	satt348	5491146–5491362	995.87	qATI10-1	Satt269	93.41	3.92
AT3G55990 (ESK1)	Glyma14g06370	14	4626260–4623046	sat_342	2954747–2954981	1,668.30	qATI14	Satt126	309.53	7.32
AT2G40950 (BZIP17)	Glyma19g30680	19	38336451–38334669	satt652	9202248–9202462	29,132.42	qSTI19	AW508247	834.98	7.95
AT3G05880 (RCI2A)	Glyma20g22290	20	32331094–32331431	satt270	34223110–34223331	1,892.02	qSTI20, qSTI20e	Satt354	1098.67	3.89

### Monte Carlo simulation studies

#### Comparison of new method with both single-QTL method and test of independence

**Figure 2:**
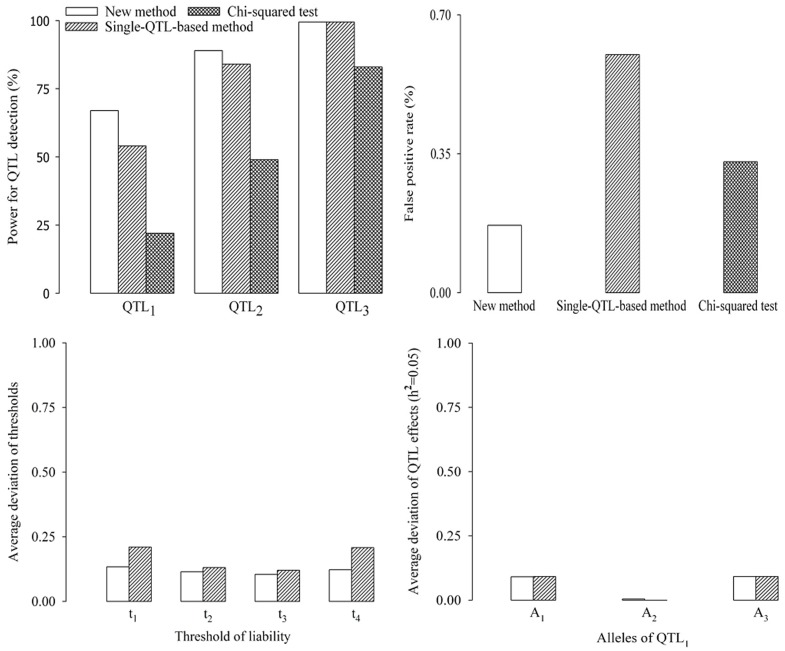
Comparison of new method with single-QTL-based method and Chi-squared test.

In the first simulation experiment, each simulated sample was analyzed by three methods. One is multi-QTL-based method in this study (new method), one is to use the new method under the condition of single-QTL model and one is test of independence. All the results are shown in **Fig. 2**. Among the three methods, the statistical power of the new method is the maximum, and the false positive rate (FPR) of the new method is the minimum. The estimates of QTL effects and threshold values from the new method are closer to the corresponding true values than those from single-QTL method, although all the estimates were slightly biased. Relatively small variations were observed in the new method for the estimates of position and effects of QTL as well as the threshold values. Therefore, the new method works relatively well.

#### Effect of phenotypic distribution on QTL mapping

**Figure 3:**
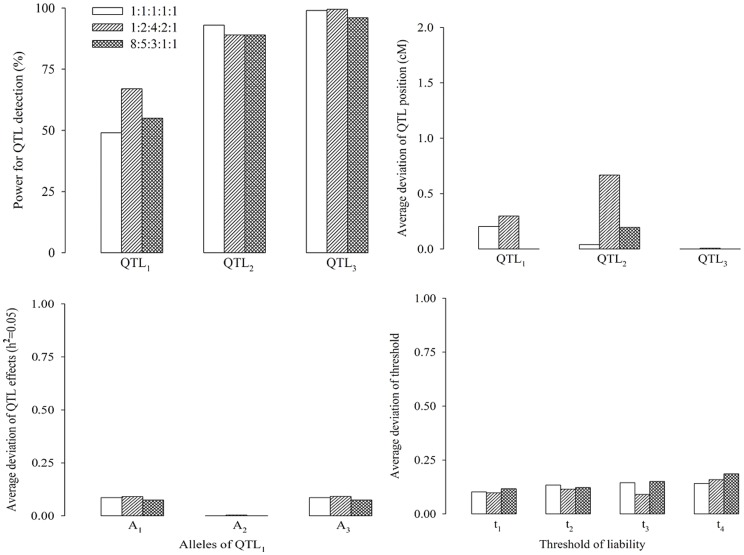
Effect of phenotypic distribution on association mapping for ordinal traits.

In the second simulation experiment, the effect of the shape of phenotypic distribution on the new method was assessed by letting the phenotypic distribution of five ordinal categories be set as 1∶1∶1∶1∶1 (uniform distribution), 1∶2∶4∶2∶1 (symmetrical distribution) and 8∶5∶3∶1∶1 (skewed distribution). Other parameters were the same as those in the first simulation experiment. The results are given in **Fig. 3**. We found that skewed distribution has decreased the statistical power. The optimal power occurred in the situation where the phenotypic distribution is bell-shaped. Relatively small variations were also observed in the three situations for the estimates of position and effects of QTL as well as the threshold values.

#### Effect of the number of categories on QTL mapping

**Figure 4:**
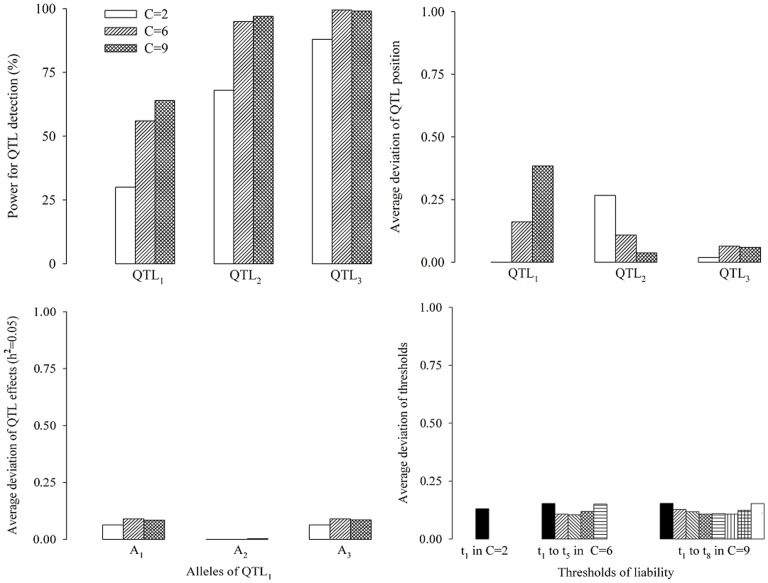
Effect of the number of categories on association mapping for ordinal traits.

In the third simulation experiment, we evaluated the effect of the number of categories on the new method. The design of the simulation was similar to that described in the first simulation experiment, except for the number of phenotypic categories. We simulated three levels for the number of categories: 2, 6 and 9. The corresponding phenotypic distributions were 1∶1, 1∶3∶6∶6∶3∶1 and 1∶2∶4∶6∶9∶6∶4∶2∶1, respectively. The results are given in **Fig. 4**, which shows that the estimate of QTL position is very close to its true value in the three cases, and the power for QTL detection increases as the number of categories increases. The reason is that increasing the number of categories has increased the information of predicting the liability from the observed categorical phenotype. In addition, relatively small variations were also observed in the three situations for the estimates of QTL effects and the threshold values.

#### Effect of sample size on QTL mapping

**Figure 5:**
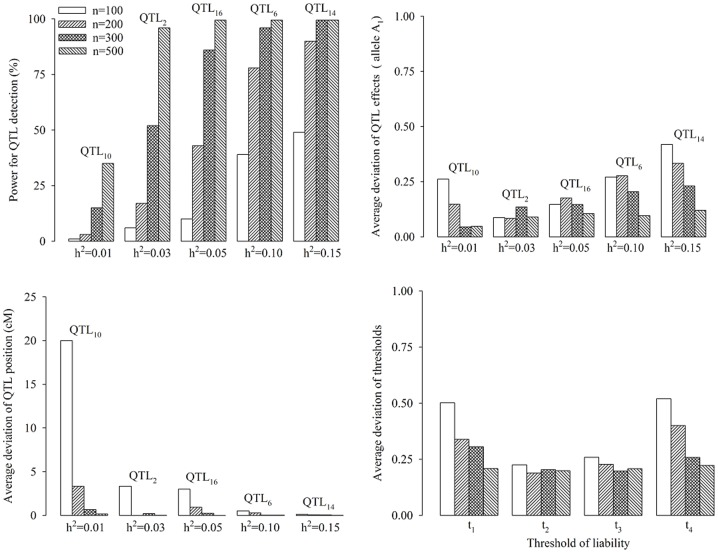
Effect of sample size on association mapping for ordinal traits.

In the fourth simulation experiment, we assumed the pedigree to have the numbers of non-founders of 100, 200, 300 and 500, and the number of founders of 50. One hundred and one equally spaced markers, each with three alleles, were placed on each of three 1000 cM chromosome segments; and eighteen QTL, each with three alleles, were simulated with heritabilities of 0.01–0.15. Other parameters were given in **Table 3**. The results of five QTL are shown in **Fig. 5**. As expected, the QTL detection power increases and the variations for the estimates of QTL parameters and the threshold values decreases as sample size or QTL heritability increases.

**Table 3:** Simulated parameters in all simulated experiments (3 alleles for marker and QTL, and 3 chromosomes).

Case	Maize pedigree		Marker Density (cM)	Genome length (cM)	Phenotype		QTL	
	No. of founders	No. of Non-founders			No. of categories	Distribution	Position (cM)	Heritability (%)
1	100	200	Equal, 10	100×3	5	1∶2∶4∶2∶1	50, 50, 50	5, 10, 15
2	100	200	Equal, 10	100×3	5	1∶1∶1∶1∶1; 1∶2∶4∶2∶1; 8∶5∶3∶1∶1	50, 50, 50	5, 10, 15
3	100	200	Equal, 10	100×3	2,6,9	1∶1; 1∶3∶6∶6∶3∶1;1∶2∶4∶6∶9∶6∶4∶2∶1	50, 50, 50	5, 10, 15
4	100	200	Equal, 10	100×3	5	1∶2∶4∶2∶1	50, 50, 50	5, 10, 15
5	50	300,200,100	Equal, 10	1000×3	5	1∶2∶4∶2:1	90,240,390,540,690,840;80,230,380,530,680,830;120,270,420,570,720,870	1×5,3×5,5×6,10, 15
6	25, 50, 75	200	Equal, 10	1000×3	5	1∶2∶4∶2∶1	90,240,390,540,690,840;80,230,380,530,680,830;120,270,420,570,720,870	1×5,3×5,5×6,10,15

#### Effect of the number of founders on QTL mapping

**Figure 6:**
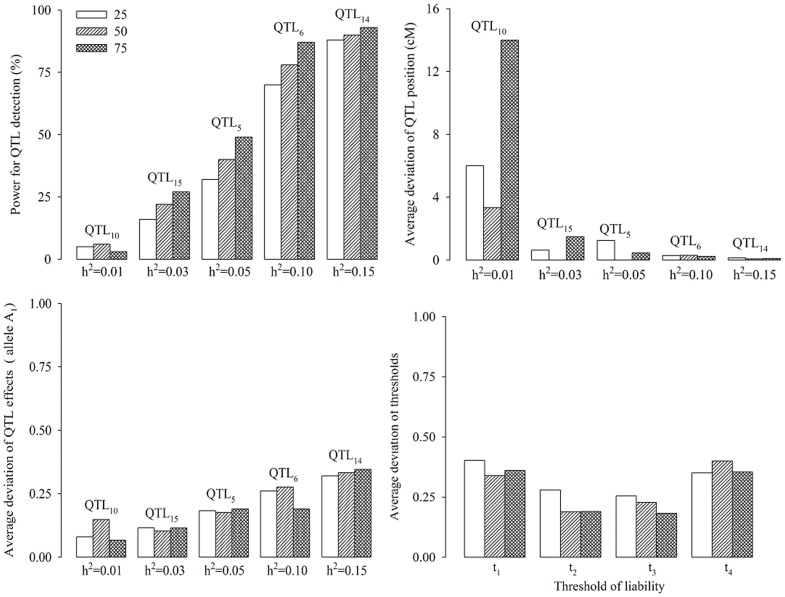
Effect of the number of founders on association mapping for ordinal traits.

In the last simulation experiment, we assumed the pedigree to have the number of non-founders of 200, and the numbers of founders of 25, 50 and 75. Other parameters were the same as those in the fourth simulation experiment. The results of five QTL are shown in **Fig. 6**. As expected, the QTL detection power increases as the founder number increases and relatively small variations and biasedness for the estimates of QTL parameters and the threshold values were observed.

## Discussion

In this study the probability of 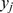, 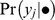, is viewed as an approximate normal distribution so that empirical Bayes approach could be adopted to estimate genetic effects in the hierarchical generalized linear model for ordinal trait association studies. As a result, M-QTL and QE interaction for ordinal traits in crop cultivars can be identified, elite alleles can be mined and novel parental combinations can be predicted. Clearly, it integrates genetic analyses with crop breeding design. More importantly, the mapping results in this study are reliable because they have been validated in four aspects. First, seven QTL detected by new method are consistent with those by at least one of three approaches: ECMLM, EAM and single marker analysis (**Table 1**). Second, a total of 7 potential candidate genes homologous to *Arabidopsis* are found to be around 7 associated markers (**Table 2**). Third, some QTL were simultaneously identified among alkaline-salt tolerance index, original and ordinal traits, for example, Sat_342 and Satt348 were associated with alkaline tolerance, and Satt270 was associated with salt tolerance. Finally, the results from Monte Carlo simulation studies show that new method improves statistical power and precision, and reduces FPR.

The major contribution of this study is the pseudo data normal approximation of the likelihood function for ordinal trait association studies. The normal likelihood approximation was first developed by Wolfinger and O'Connell [29] and continued by Gelman et al [30]. McGilchrist [31] used a different approach for the same problem, but much easier to understand. Although the method has been explored for binary and binomial traits in linkage studies [32], this study is the first report of the pseudo data approximation for ordinal trait association studies.

We compared the new method with that of Lü et al. [18]. There are some commons between the two approaches. For example, the similar effects of phenotypic distribution (the number of categories, sample size and heritability) on QTL mapping in homozygous cultivars are observed. However, the differences exist as well. For example, the trait is quantitative in Lü et al. [18] and ordinal in this study; and the power for the detection of QTL is lower for this study than for Lü et al. [18], because limited information is observed for ordinal traits. As the number of categories increases, it is better to use the normal trait hierarchical linear mixed model. Note that the main benefit of this study comes from small number of categories. Although Iwata et al. [23] and Hoggart et al. [24] are for ordinal traits, in this study main-QTL, environmental effect and QTL-by-environment interactions were simultaneously considered in our full genetic model, improving the statistical power and estimation precision.

As compared with genome-wide association studies in Yu et al. [16] and Zhang et al. [17], kinship matrix was not considered in this study. In fact, this term is related to background control, which is similar to co-variable markers in composite interval mapping. Note that all the main-effect QTL and QTL-by-environment interactions are included in the full genetic model of this study. Thus, it is unnecessary to consider this term in the current study. In addition, in real data analysis we also consider the effect of population structure on association studies. As a result, a slightly different result is observed while Q matrix is deleted from the above full model.

Epistasis, the interaction between QTL, plays an important role in the dissection of genetic architecture for complex traits. To date, several approaches have been developed, including multiple interval mapping, Bayesian approach, and penalized maximum likelihood method. Most of these methods are for quantitative traits in bi-parental segregating populations. In homozygous cultivars, it is relatively difficult. Because of its complexity, it will be investigated separately in a future project.

## Materials and Methods

### Soybean samples

257 soybean cultivars used in this study were mainly provided by the National Center for Soybean Improvement, China. All the cultivars were obtained by stratified random sampling from six geographic ecotypes in China, planted in three-row plots in a completely randomized design and evaluated at the Jiangpu experimental station at Nanjing Agricultural University in 2009 and 2010. The plots were 1.5 m wide and 2 m long. Twelve seeds for each cultivar were sown in a 30×20×15 cm plastic container with the 3.5 cm height sand and then treated with control (CK), 100 mM NaCl and 10 mM Na_2_CO_3_, and each with two replications. They were grown in a growth chamber under white fluorescent light (600 µmol m^−2^ s^−1^; 14 h light/10 h dark) at 25±1°C. Length of main root (LR, centimetre) for healthy seedlings were measured from 5 plants 7 days after sowing. To measure the degree of salt-alkaline tolerance, original trait observations might be transferred into salt-alkaline tolerance index for each trait using the below equations
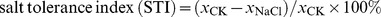


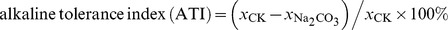
where 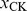,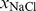 and 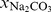 stand for phenotypic values in control, saline and alkaline treatments, respectively [33]. The tolerance grades 1 to 5, used in this study, were indicated by 0–20%, 20–40%, 40–60%, 60–80% and 80–100%, respectively.

Approximately 0.3 g of fresh leaves obtained in 2008 from each cultivar was used to extract genomic DNA using the cetyltrimethylammonium bromide method as described by Lipp et al. [34]. To screen for polymorphisms among all the cultivars, polymerase chain reaction (PCR) was performed with 135 simple sequence repeat (SSR) primer pairs. The primer sequences were obtained from the soybean database Soybase (http://www.ncbi.nlm.nih.gov). PCR was performed as described by Xu et al. [35] and Wei et al. [36].

### Population structure

For the soybean sample, the STRUCTURE software [37] was used to investigate the population structures of all selected cultivars. The number of subpopulations (*K*) was set from 2 to 10. In the Markov chain Monte Carlo (MCMC) Bayesian analysis for each *K*, the length of a Markov chain consisted of 110,000 sweeps. The first 10,000 sweeps (the burn-in period) were deleted, and thereafter, the chain was used to calculate the mean of log-likelihood. This process was repeated 20 times, and the total average for mean log-likelihood at fixed *K* was used. STRUCTURE analysis with 135 SSR molecular markers showed that the log-likelihood increased with the increase of the model parameter *K*, so a suitable number of *K* could not be determined. In this situation, using the ad hoc statistic Δ*K*, based on the rate of change in the log-probability of data between successive *K* values [38], STRUCTURE accurately detected the uppermost hierarchical level of structure. Here, the Δ*K* value was much higher for the model parameter *K* = 4 than for other values of *K* [33]. By combining this high Δ*K* value with knowledge of the breeding history of these cultivars, we chose a value of 4 for *K*. The *Q* matrix was calculated based on SSR markers and incorporated into the hierarchical generalized linear mixed model in this study.

### Generalized linear mixed Model

Let 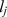 (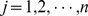) be the vector of underlying latent variable or liability of cultivar *j*. For the *j*th cultivar, it is postulated that
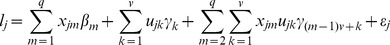
(1)where 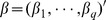 is non-genetic effects, i.e., population mean (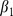) and environmental effect 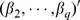; 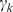 is allelic effect for 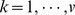 and allele-by-environment interaction effect for 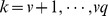, 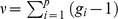, and 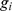 is the number of alleles for locus 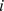 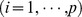; 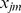 and 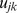 are dummy variables of 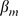 and 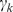 for cultivar *j*, respectively; and 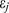 is the random residual error with an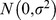 distribution. 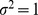 will be adopted here because the liabilities are unobservable.

Methods of estimating allelic effects and allele-by-environment interaction effects are the same. For the sake of clarity of notation, we redefine the design matrix and the regression coefficients as follows. Let 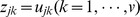 and 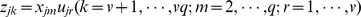. The above model is now rewritten as
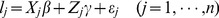
(2)where 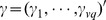.

Let 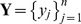 denote the vector of observed ordinal data. Here each 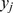 represents an assignment into *C* ordinal categories. These classes result from the hypothetical existence of 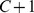 thresholds (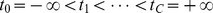) in the latent scale. The relationship between 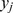 and 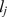 is indicated by
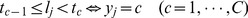
(3)


The conditional probability that 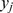 falls in category *c*, given 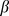, 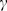 and 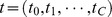, is given by
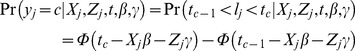
(4)where 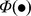 is the cumulative distribution function of standard normal distribution. The data are conditionally independent, given 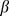, 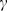 and 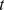. Therefore, log-likelihood function can be written as
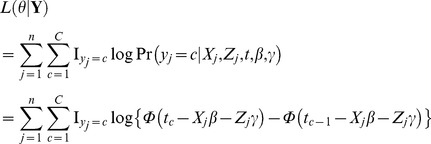
(5)where 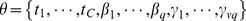; and 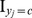 is an indicator function taking value of 1 if 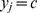 and 0 otherwise.

### Prior distribution and joint posterior density

The parameters 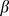 and 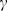 are treated as fixed and random effects, respectively. The number of random effects in the above genetic model is very large so that the model is oversaturated. Therefore, the hierarchical generalized linear mixed model is adopted in this study. It is assumed that each genetic effect 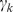 has a different variance 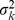. The following prior distributions are chosen for building the hierarchical model
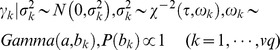
where 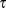 and 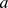 are the constants given in advance. When 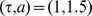, the method works well. The joint posterior distribution has a form of
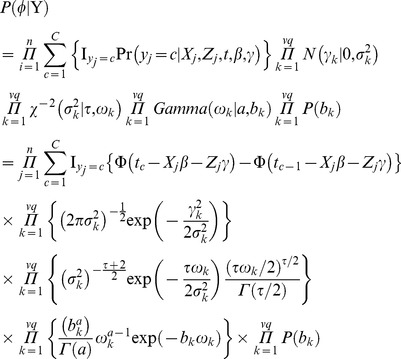
(6)where 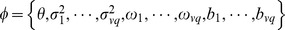


### Parameter estimation

#### Genetic effect

As shown in Wolfinger and O'Connell [29], 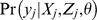 is an approximate normal distribution 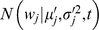, where pseudo-data 
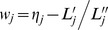
, 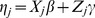; pseudo-mean 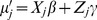; pseudo-variance 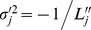; 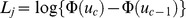, 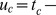 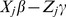; 
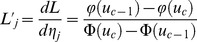
; 
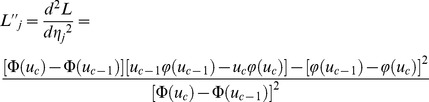
where 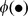 is the probability density function of standard normal distribution. The conditional log-posterior distribution, related to 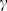, is indicated by
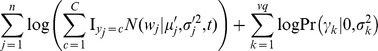



Using expectation-maximization empirical Bayes approach of Xu [26], the expectation of the quadratic term required in the maximization step is expressed as
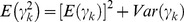
(7)where 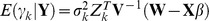, 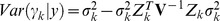, 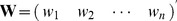, 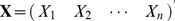, and 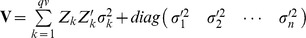. Once a certain criterion of convergence is satisfied, the converged 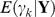 is the estimate for 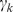.

#### Genetic effect variances and related hyperparameters

According to joint posterior density in equation (6), conditional posterior distribution is 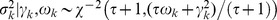 for 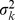, 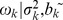 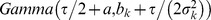 for 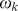 and 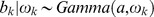 for 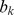. Here the mode is used to estimate the corresponding parameter, such as,
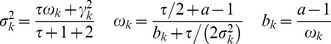
(8)


#### Non-genetic effect β

Formula for the fixed effect follows the standard procedure of mixed model methodology, we have
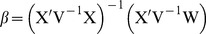
(9)


#### Thresholds

Using the Newton–Raphson method, the threshold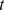 are estimated by
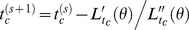
(10)where 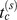 is the estimate of parameter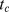 at the *s*th iteration,
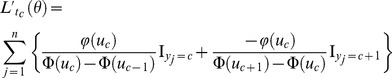


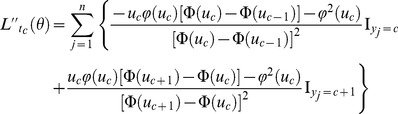



### Summary of iterations

1. Let 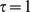 and 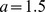, and provide initial values for 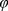, for example, let 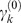 be an uniform random number, 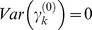, 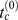 be the quantile of the standard normal distribution based on the phenotypic distribution of 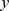, 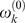 be a gamma random number. 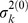 and 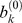 can be obtained by equation (8).

2. Update 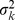, 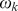 and 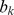 using equation (8);

3. Update 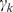 using the estimate of 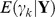;

4. Update 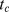 using equation (10);

5. Update 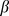 using equation (9);

6. Repeat step 2 to step 5 until predetermined criterion of convergence is satisfied.

### Statistical test

A two-stage selection process in Lü et al. [18] was used to conduct likelihood ratio test (LRT) for all the QTL. In the first stage, all the markers were included in the model. If the estimate of an absolutely allelic effect (environmental interaction effect) at the *k*th locus 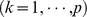 is greater than 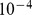, the *k*th locus is picked up. In the second stage, we modified the full model only to contain the effects passing the first round of selection. If doing so, we can use the maximum likelihood method to perform the LRT.

The overall null hypothesis is no effect of the *o*th QTL (or interacted QTL), denoted by 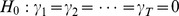, where 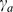 is the *a*th effect for the QTL. If we solve the maximum likelihood estimation of the parameters under the restriction of the 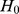 and calculate the log-likelihood value using the solutions with this restriction, we obtain 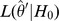. We can also evaluate the log-likelihood value of the solutions without restrictions and obtain 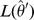. Therefore, the LRT statistic is 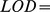 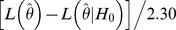 and the significance threshold of the LOD score was set at 2.0.

### Simulation design

We performed six simulation experiments in this study. In the first, the simulated pedigree was similar to the maize pedigree described by Zhang et al. [15]. In current pedigree, the numbers of founders and non-founders were 100 and 200, respectively. Of these, founder lines were in linkage equilibrium so that the genotypes for markers and QTL with three alleles could be simulated. In other words, three alleles for each locus were assigned in equal proportions to each founder. Non-founders were bred via repeated self-pollination of a hybrid between two inbred lines. Thus, each non-founder line represents a RIL with respect to a known pair of parents. The genotypes of all the non-founders could be generated from the genotypes of their parents, analogous to simulating the genotypes of RIL from their parents. All of the non-founder lines could be used to detect QTL. Thirty-three equally spaced markers were simulated on three-chromosome segments 300 cM long. A total of 3 QTL, all of which overlapped with the markers, were placed at 50 cM of each chromosome; the QTL size, being the proportion of total phenotypic variance explained by the QTL, is 0.05, 0.10 and 0.15, respectively. The allelic effects were calculated by relating the genetic variance of the QTL to the allelic frequencies and effects. The phenotypic value of each line was the sum of the corresponding QTL genotypic values and the residual error, with an assumed normal distribution. These phenotypic values could be transferred into five ordinal categories with four threshold values: −1.2816, −0.5244, 0.5244 and 1.2816. Therefore, the frequencies of the five ordinal categories occurring in all the inbred lines have a ratio of 1∶2∶4∶2∶1. Each simulation run consisted of 100 replicates. For each simulated QTL, we counted the samples in which the LOD statistic surpassed 2.0. The ratio of the number of such samples (m) to the total number of replicates (100) represented the empirical power of this QTL. The FPR was calculated as the ratio of the number of false positive effects to the total number of zero effects considered in the full model. The other simulation experiments were performed similarly. All simulated parameters are given in **Table 3**.

A SAS program is available from the authors on request.

## Supporting Information

Table S1 Phenotypic values of ATI and STI in 257 soybean cultivars under study.
(DOC)
