# Supplementary material for: An Efficient Hierarchical Generalized Linear Mixed Model for Mapping QTL of Ordinal Traits in Crop Cultivars
Source: PLoS One. 2013 Apr 2;8(4):e59541. doi: 10.1371/journal.pone.0059541 (PMC3614919; doi:10.1371/journal.pone.0059541)
Supplement: Table S1 — Phenotypic values of ATI and STI in 257 soybean cultivars under study. (DOC) [file pone.0059541.s001.doc]

**Table S1. Phenotypic values of ATI and STI in 257 soybean cultivars under** study

| No. | ATI | | STI | | No. | ATI | | STI | | No. | ATI | | STI | | No. | ATI | | STI | |
| --- | --- | --- | --- | --- | --- | --- | --- | --- | --- | --- | --- | --- | --- | --- | --- | --- | --- | --- | --- |
|  | 2009 | 2010 | 2009 | 2010 |  | 2009 | 2010 | 2009 | 2010 |  | 2009 | 2010 | 2009 | 2010 |  | 2009 | 2010 | 2009 | 2010 |
| 1 | 3 | 2 | 3 | 3 | 66 | 2 | 1 | 3 | 2 | 131 | 3 | 3 | 3 | 3 | 196 | 2 | 2 | 2 | 3 |
| 2 | 2 | 3 | 4 | 3 | 67 | 2 | 3 | 2 | 3 | 132 | 2 | 1 | 2 | 3 | 197 | NA | 1 | NA | 3 |
| 3 | 2 | 2 | 3 | 2 | 68 | 1 | 1 | 1 | 2 | 133 | NA | 2 | NA | 3 | 198 | 5 | 1 | 2 | 2 |
| 4 | 2 | 1 | 3 | 2 | 69 | 2 | 2 | 3 | 3 | 134 | NA | 1 | NA | 2 | 199 | 2 | 1 | 3 | 3 |
| 5 | NA | 2 | NA | 3 | 70 | 2 | 1 | 3 | 2 | 135 | 2 | 1 | 2 | 3 | 200 | 3 | 1 | 3 | 3 |
| 6 | 2 | 2 | 2 | 2 | 71 | 2 | 2 | 3 | 3 | 136 | NA | 1 | NA | 3 | 201 | 2 | 1 | 3 | 3 |
| 7 | 1 | 1 | 2 | 3 | 72 | 3 | 1 | 3 | 3 | 137 | 3 | 2 | 3 | 3 | 202 | 1 | 2 | 3 | 3 |
| 8 | 1 | 2 | 1 | 3 | 73 | 2 | 1 | 2 | 3 | 138 | 2 | 2 | 2 | 3 | 203 | 2 | 2 | 3 | 3 |
| 9 | 2 | 2 | 3 | 3 | 74 | 2 | 2 | 3 | 2 | 139 | 3 | 3 | 3 | 3 | 204 | NA | 1 | NA | NA |
| 10 | 3 | 2 | 3 | 3 | 75 | 2 | 3 | 3 | 3 | 140 | 2 | 2 | 3 | 3 | 205 | NA | 2 | NA | 3 |
| 11 | 2 | 1 | 3 | 3 | 76 | 3 | 1 | 3 | 3 | 141 | 2 | 2 | 3 | 2 | 206 | 4 | 2 | NA | 2 |
| 12 | 2 | 1 | 3 | 3 | 77 | 2 | 2 | 2 | 3 | 142 | 3 | 4 | 4 | 3 | 207 | 3 | 3 | 4 | 3 |
| 13 | 3 | 2 | 3 | 3 | 78 | 2 | 2 | 3 | 2 | 143 | 2 | 2 | 2 | 2 | 208 | 2 | 1 | 3 | 2 |
| 14 | 2 | 2 | 3 | 3 | 79 | 3 | 2 | 3 | 3 | 144 | 2 | 2 | 3 | 3 | 209 | 2 | 1 | 3 | 3 |
| 15 | 2 | 1 | 2 | 2 | 80 | 3 | 2 | 3 | 3 | 145 | 2 | 2 | 2 | 3 | 210 | 2 | 1 | 2 | 2 |
| 16 | 2 | 1 | 4 | 2 | 81 | 2 | 2 | 4 | 3 | 146 | 3 | 1 | 3 | 3 | 211 | 2 | 1 | 3 | 3 |
| 17 | 2 | NA | 3 | NA | 82 | 3 | 1 | 3 | 3 | 147 | 2 | 1 | 1 | 1 | 212 | 1 | NA | 2 | NA |
| 18 | NA | NA | NA | 2 | 83 | 1 | 1 | 2 | 3 | 148 | 1 | 2 | 2 | 3 | 213 | 1 | 1 | 3 | 3 |
| 19 | 2 | 2 | 3 | 3 | 84 | 2 | NA | 2 | NA | 149 | 3 | 1 | 3 | 3 | 214 | 2 | 3 | 2 | 3 |
| 20 | 3 | 1 | 3 | 3 | 85 | 1 | 1 | 3 | 3 | 150 | 2 | 2 | 3 | 3 | 215 | 1 | 2 | 3 | 3 |
| 21 | 2 | 1 | 3 | 3 | 86 | 2 | 1 | 2 | 3 | 151 | 2 | 2 | 3 | 3 | 216 | 1 | NA | 2 | 3 |
| 22 | 3 | 2 | 3 | 3 | 87 | 1 | 1 | 2 | 2 | 152 | 2 | 1 | 2 | 3 | 217 | 3 | 1 | 3 | 3 |
| 23 | 1 | 2 | 2 | 3 | 88 | 1 | 2 | 1 | 3 | 153 | 2 | 1 | 2 | 3 | 218 | 2 | 2 | NA | 3 |
| 24 | 1 | 1 | 2 | 2 | 89 | 2 | 4 | 3 | 3 | 154 | 1 | 1 | 3 | 4 | 219 | 3 | 1 | 3 | 3 |
| 25 | NA | 2 | NA | 3 | 90 | 2 | 1 | 3 | 3 | 155 | 2 | 1 | 2 | 2 | 220 | 2 | 2 | 3 | 2 |
| 26 | 3 | 2 | 4 | 3 | 91 | 3 | 2 | 2 | 3 | 156 | 3 | 3 | 3 | 4 | 221 | 2 | 1 | 2 | 3 |
| 27 | 2 | 1 | 2 | 2 | 92 | 1 | 2 | 2 | 4 | 157 | 2 | 1 | 3 | 2 | 222 | NA | 2 | NA | NA |
| 28 | 1 | 2 | 2 | 3 | 93 | 5 | 3 | 3 | 3 | 158 | 3 | 1 | 3 | 3 | 223 | 2 | 1 | 3 | 1 |
| 29 | 3 | 1 | 4 | 2 | 94 | 1 | 1 | 2 | 2 | 159 | 1 | 1 | 1 | 2 | 224 | 1 | 1 | 3 | 2 |
| 30 | 2 | 2 | 3 | 4 | 95 | 1 | 1 | 2 | 2 | 160 | 2 | 1 | 2 | 3 | 225 | 3 | 2 | 4 | 3 |
| 31 | NA | 1 | NA | 2 | 96 | 2 | 1 | 3 | 3 | 161 | 1 | 1 | 2 | 3 | 226 | 1 | 2 | 2 | NA |
| 32 | 3 | 2 | 3 | 3 | 97 | 3 | 2 | 3 | 3 | 162 | 3 | 2 | 3 | 3 | 227 | 3 | 3 | 3 | 3 |
| 33 | 1 | 1 | 2 | 3 | 98 | 3 | 3 | 3 | 3 | 163 | 3 | 2 | 3 | 3 | 228 | 2 | 1 | 3 | 3 |
| 34 | 1 | 1 | 1 | 3 | 99 | 4 | 1 | 3 | 3 | 164 | 2 | 1 | 3 | 4 | 229 | 2 | 2 | 2 | 3 |
| 35 | 1 | 3 | 1 | 3 | 100 | 3 | 3 | 3 | 3 | 165 | 2 | 1 | 2 | 2 | 230 | NA | 2 | NA | NA |
| 36 | 3 | 1 | 3 | 3 | 101 | 5 | 3 | 3 | 3 | 166 | 2 | 2 | 2 | 3 | 231 | 2 | 1 | 3 | 3 |
| 37 | 1 | 2 | 3 | 2 | 102 | NA | 2 | NA | 3 | 167 | 2 | 2 | 3 | 3 | 232 | 4 | 1 | 4 | 3 |
| 38 | 3 | NA | 3 | NA | 103 | 2 | 1 | 3 | 2 | 168 | 4 | 2 | 4 | 2 | 233 | 2 | 1 | 3 | 2 |
| 39 | 3 | NA | 4 | NA | 104 | 3 | 1 | 3 | 2 | 169 | 2 | 1 | 3 | NA | 234 | 3 | 2 | 3 | 3 |
| 40 | NA | 2 | NA | NA | 105 | 2 | 1 | 3 | 3 | 170 | NA | 1 | NA | 3 | 235 | 3 | 2 | 3 | 3 |
| 41 | 1 | 2 | 1 | 3 | 106 | NA | 1 | NA | NA | 171 | 1 | 2 | 2 | 3 | 236 | 2 | 2 | 2 | 4 |
| 42 | NA | 3 | NA | 2 | 107 | 1 | NA | 2 | 2 | 172 | 1 | 2 | 2 | 3 | 237 | 1 | 2 | 2 | 3 |
| 43 | 4 | 1 | 4 | 1 | 108 | 1 | 2 | 2 | 3 | 173 | 3 | 2 | NA | 3 | 238 | NA | 2 | NA | 3 |
| 44 | 2 | 2 | 3 | 3 | 109 | 1 | 1 | 3 | 3 | 174 | 2 | 1 | 2 | 3 | 239 | 2 | 2 | 3 | 3 |
| 45 | 2 | 2 | 2 | 3 | 110 | 1 | NA | 2 | NA | 175 | 1 | 1 | 3 | 3 | 240 | 3 | 1 | 3 | 3 |
| 46 | 1 | 1 | 3 | 3 | 111 | 1 | 2 | 2 | 2 | 176 | 1 | 1 | NA | 2 | 241 | 1 | 4 | 3 | 3 |
| 47 | 3 | 2 | 3 |  | 112 | 2 | 1 | 3 | 3 | 177 | 3 | 2 | 3 | 3 | 242 | 1 | 1 | 3 | NA |
| 48 | 3 | 1 | 3 | 3 | 113 | 4 | 2 | 3 | 2 | 178 | 3 | NA | 3 | NA | 243 | 2 | 4 | 3 | 3 |
| 49 | 2 | 3 | 3 | 3 | 114 | 3 | 1 | 3 | 3 | 179 | 2 | 2 | 2 | 3 | 244 | 1 | 1 | 3 | NA |
| 50 | 1 | 1 | 2 | 2 | 115 | 2 | 1 | 3 | 3 | 180 | 3 | NA | 3 | NA | 245 | 3 | 1 | 3 | 3 |
| 51 | 1 | 2 | 2 | 3 | 116 | 5 | 3 | 3 | 3 | 181 | 2 | 2 | 3 | 3 | 246 | 2 | 2 | 3 | 3 |
| 52 | 2 | 1 | 3 | 3 | 117 | 2 | 2 | 3 | 3 | 182 | NA | 3 | NA | 3 | 247 | 2 | 1 | 2 | 3 |
| 53 | 2 | 2 | 3 | 3 | 118 | 2 | 1 | 3 | 3 | 183 | 3 | 2 | 3 | 3 | 248 | 3 | 2 | 3 | 3 |
| 54 | 1 | 1 | 3 | 2 | 119 | 2 | 1 | 2 | 2 | 184 | 4 | 3 | 3 | 3 | 249 | 2 | 3 | 2 | 3 |
| 55 | 1 | 2 | 2 | 2 | 120 | 2 | 2 | 2 | 3 | 185 | 2 | 2 | 3 | NA | 250 | 3 | NA | 3 | 2 |
| 56 | 2 | 3 | 2 | 3 | 121 | 3 | 2 | 3 | 3 | 186 | 4 | 2 | 4 | 4 | 251 | 2 | 1 | 3 | 2 |
| 57 | NA | 2 | NA | 4 | 122 | 1 | 1 | 1 | 3 | 187 | 2 | NA | 3 | NA | 252 | 3 | NA | 4 | NA |
| 58 | 2 | 1 | 2 | 3 | 123 | 2 | 2 | 3 | 3 | 188 | 2 | 1 | 3 | 3 | 253 | 3 | 3 | 3 | 3 |
| 59 | 4 | 2 | 3 | 3 | 124 | 4 | 2 | 3 | 3 | 189 | 2 | 2 | 2 | 3 | 254 | 2 | 2 | 2 | 3 |
| 60 | 2 | 1 | 2 | 3 | 125 | 2 | 1 | 3 | 3 | 190 | 2 | 1 | 4 | 2 | 255 | 2 | 3 | 3 | 3 |
| 61 | 1 | 2 | 2 | 3 | 126 | 3 | 1 | 3 | 3 | 191 | 2 | 1 | 4 | 2 | 256 | 3 | 1 | 3 | 3 |
| 62 | 2 | 1 | 3 | 2 | 127 | 1 | 1 | 3 | 2 | 192 | 3 | 1 | 3 | 3 | 257 | 3 | 2 | 2 | 3 |
| 63 | 1 | 1 | 2 | 3 | 128 | 3 | 1 | 3 | 3 | 193 | 2 | 2 | 3 | 3 |  |  |  |  |  |
| 64 | 1 | 1 | 2 | 3 | 129 | 1 | 1 | 1 | 2 | 194 | NA | 1 | NA | 3 |  |  |  |  |  |
| 65 | 3 | 2 | 4 | 3 | 130 | 2 | 2 | 3 | 3 | 195 | 2 | 2 | 3 | 3 |  |  |  |  |  |

Salt-alkaline tolerance indexes in 2009 and 2010 were partitioned into five grades: 1 (high tolerance), 2 (tolerance), 3 (middle tolerance), 4 (sensitivity), and 5 (high sensitivity). NA: no available.
